# The Application of Classification and Regression Trees for the Triage of Women for Referral to Colposcopy and the Estimation of Risk for Cervical Intraepithelial Neoplasia: A Study Based on 1625 Cases with Incomplete Data from Molecular Tests

**DOI:** 10.1155/2015/914740

**Published:** 2015-08-03

**Authors:** Abraham Pouliakis, Efrossyni Karakitsou, Charalampos Chrelias, Asimakis Pappas, Ioannis Panayiotides, George Valasoulis, Maria Kyrgiou, Evangelos Paraskevaidis, Petros Karakitsos

**Affiliations:** ^1^Department of Cytopathology, University of Athens Medical School, Attikon University Hospital, Rimini 1, Haidari, 12462 Athens, Greece; ^2^Biomedical Engineering Laboratory, National Technical University of Athens, Iroon Politechniou 9, Zografou, 15780 Athens, Greece; ^3^3rd Department of Obstetrics and Gynecology, University of Athens Medical School, Attikon University Hospital, Rimini 1, Haidari, 12462 Athens, Greece; ^4^2nd Department of Pathology, University of Athens Medical School, Attikon University Hospital, Rimini 1, Haidari, 12462 Athens, Greece; ^5^Department of Obstetrics & Gynecology, University Hospital of Ioannina, Stavros Niarchos Avenue, 45500 Ioannina, Greece; ^6^Department of Obstetrics & Gynaecology, Worthing Hospital, Western Sussex Hospitals NHS Foundation Trust, Worthing BN11 2DH, UK; ^7^Department of Surgery and Cancer, Institute of Reproductive & Developmental Biology, Faculty of Medicine, Imperial College, London W12 0NN, UK; ^8^West London Gynaecological Cancer Center, Queen Charlotte's and Chelsea-Hammersmith Hospital, Imperial Healthcare NHS Trust, London W12 0HS, UK

## Abstract

*Objective*. Nowadays numerous ancillary techniques detecting HPV DNA and mRNA compete with cytology; however no perfect test exists; in this study we evaluated classification and regression trees (CARTs) for the production of triage rules and estimate the risk for cervical intraepithelial neoplasia (CIN) in cases with ASCUS+ in cytology. *Study Design*. We used 1625 cases. In contrast to other approaches we used missing data to increase the data volume, obtain more accurate results, and simulate real conditions in the everyday practice of gynecologic clinics and laboratories. The proposed CART was based on the cytological result, HPV DNA typing, HPV mRNA detection based on NASBA and flow cytometry, p16 immunocytochemical expression, and finally age and parous status. *Results*. Algorithms useful for the triage of women were produced; gynecologists could apply these in conjunction with available examination results and conclude to an estimation of the risk for a woman to harbor CIN expressed as a probability. *Conclusions*. The most important test was the cytological examination; however the CART handled cases with inadequate cytological outcome and increased the diagnostic accuracy by exploiting the results of ancillary techniques even if there were inadequate missing data. The CART performance was better than any other single test involved in this study.

## 1. Introduction

Cervical cancer (CC) is the third most common cancer and the fourth leading cause of cancer death in females worldwide [1]. More than 85% of these cases and deaths are in developing countries; this is due to lack of screening that may allow detection of precancerous and early stage cervical cancer. Despite the advances in screening, cervical cancer remains a serious problem of public health even in developed countries, due to the high percentage of detection failures [2].

CC is known to be caused almost always by human papillomavirus (HPV) infection which is the commonest sexually transmitted infection worldwide. About 100 types of HPV virus that can infect humans have been identified. Among them, 15 are oncogenic and can cause CC. Improved understanding of HPV infection and the natural history of cervical neoplasia have resulted in the addition of the HPV DNA test along with the Pap test and frequently a competing test.

Nowadays, ancillary techniques for CC screening are available. These include HPV DNA typing and mRNA identification of the viral E6/E7 oncogenes that are linked to oncogenic activation. Among them, mRNA typing with nucleic acid based amplification (NASBA) [3] and mRNA-Flow-FISH techniques in screening programs produced promising results in increasing PPV and reducing unnecessary recalls and referrals to colposcopy [4–7]. At the same time, it was reported that the immunocytochemical detection of p16 can increase the diagnostic accuracy of the Pap test [8].

Several published studies in the literature attempted to clarify the role of each technique as a unique test to substitute the Pap test [3–7, 9–17]. By the detailed analysis of these, it can be concluded that the performance measures of the methods under control differ significantly, affected by the disease incidence and the prevalence of HPV infection in the population study group; thus, application of a single method, even if it offers a level of protection, does not determine reliably the risk for individual women to harbor cervical intraepithelial neoplasia (CIN). However, from the meta-analysis of published studies [9–12] it is evident that the sensitivity of Pap test combined with the HPV DNA test is higher than the sensitivity of each individual method.

Computer science and artificial intelligence enabled the development of computer assisted systems for the support of clinical diagnosis or therapeutic and treatment decisions. Various classification techniques such as neural networks [18–29], discriminant analysis [18, 30–32], classification and regression trees (CARTs) [33–35], or genetic algorithms [36] have been used in medicine. The application of new molecular techniques that are nowadays used in the diagnostic cytology laboratory [37] improves the accuracy of the final diagnosis in comparison to that of cytology alone.

Among the various decision support techniques (CARTs) is an attractive statistical approach to extract knowledge from data as they are straightforward to construct and easily understandable by physicians. The application of these systems produces simple decision algorithms linked with probabilities that can be promising to define triage rules and perhaps give a better understanding of the disease.

The aim of this study was to investigate the potential role of CARTs applied on various diagnostic variables measured in the modern cytopathology laboratory and to build algorithms for the triage of individual cases. Special focus was given to design the study as pragmatic as possible: thus, (1) cases from two different parts of the country were selected, (2) a part of the cases were considered to be negative as no histological confirmation could be obtained due to ethical reasons, and (3) inadequate test results (i.e., missing data) were included as this is the cytopathology laboratory reality.

## 2. Materials and Methods

### 2.1. Involved Institutes and Ethics

Our study involved the 3rd Department of Obstetrics and Gynecology, the Department of Cytopathology and the 2nd Department of Pathology, all three hosted in “Attikon” University Hospital, Medical School of Athens University, and the Department of Obstetrics and Gynecology of University Hospital of Ioannina City. The study was approved by the University Hospital Ethics Boards and participating women signed an informed consent (ICON) form to allow use of their epidemiologic, diagnostic, and ancillary test data.

### 2.2. Cytology

All cytological and ancillary examinations were based on ThinPrep liquid based cytology (LBC) material obtained before colposcopical examination. The smears were routinely prepared for cytological examination and the remaining material in the ThinPrep vial was used for additional evaluation of biomarkers related to the HPV lifecycle. The smears were assessed by experienced cytopathologists. Histological material was obtained during colposcopy and/or during treatment by conization. The obtained histological samples were fixed and prepared according to standard histopathology protocols.

The cytological findings for each woman were formulated according to the revised Bethesda classification system (TBS2001 system) [38, 39].

### 2.3. Histological Confirmation

The histological diagnosis was the golden standard and was used as the target category of each woman. Punch biopsies were performed by experienced colposcopists (in practice for more than 10 years) as part of the study protocol. The three-tiered cervical intraepithelial neoplasia grading system was used for reporting histological diagnosis. Clinically negative (CN) cases were included in the study. These were defined as CN if the cytology, colposcopy, and the CLART Human Papillomavirus 2 HPV DNA test (see Section 2.4) were all negative. Despite the lack of histological biopsies due to ethical hurdles, these women were included and analyzed in a target category of less than CIN2. The correlation of the cytological results in relation to histology is presented in Table 1.

### 2.4. Molecular Tests

In relation to the HPV lifecycle biomarkers we used (a) HPV DNA typing using the CLART Human Papillomavirus 2 (GENOMICA) kit for the simultaneous detection of 35 different HPV genotypes by PCR amplification of a fragment within the highly conserved L1 region of the virus [40]; (b) NASBA assays [41] (NucliSENS EasyQ HPV v1.0) that were used for the identification of E6/E7 mRNA of the HPV types: 16, 18, 31, 33, and 45; (c) the PermiFlow (Invirion Diagnostics, LLC, Oak Brook, IL) kit for the identification of E6/E7 mRNA expression of high risk HPV using flow cytometry [6]; and (d) the immunocytochemical expression of p16 using the CINtec Cytology Kit [42]. In addition to pure medical data, epidemiologic features were involved as well, specifically woman age and parous status.

Within the clinical laboratory, it is not infrequent that an ancillary test produces invalid results or the biological material that remains in the vial is not adequate to perform additional tests; therefore, it is not guaranteed that there are available sets of such data for all women participating in the study. Additionally parous details were not available for all women as such data often were not considered important and referral forms were incomplete.

### 2.5. Golden Standard

Our target was to classify each woman into one of the following categories: (a) <CIN2, which included the histologically negative and CIN-1 cases as well as the CN cases, and (b) ≥CIN2, which included the histological categories: CIN-2, CIN-3, SCC, and ADENO-CA.

### 2.6. Data Formulation

For each case, a vector of 50 variables was created (Table 2); this had the result of the cytological examination expressed according to the Bethesda system. Results of the HPV DNA test examination were expressed as 35 individual values (either positive or negative), one for each HPV DNA genotype; additionally, in relation to the found subtypes five other variables were investigated: the existence of high risk, low risk, or any type as well as the number of high risk and the number of low risk types that were identified. For the NASBA HPV mRNA typing, we used the result for each individual HPV type (16, 18, 31, 33, and 45). The result of the PermiFlow test was involved using two methods, either as a percentage or as positive or negative (the cut-off value to assign a flow cytometry result was 1.5%); in addition, the result of the immunocytochemical expression of p16 was included. Finally, two other variables were entered to the tree construction process: woman age and parous status. For all variables, if there were no data, the value was left blank or declared inadequate indicating that there was no valid result or there was not adequate material in the ThinPrep vial to perform additional examinations.

### 2.7. Statistical Techniques and Modeling

The CART model was created using IBM SPSS Statistics 19 for Windows (SPSS Inc., Chicago, USA). The CART algorithm is possible to be configured and use a specific feature at the first node of the tree; however, in this study CART was allowed to select as first test the test with the highest overall accuracy. To assess the performance, various statistical measures were extracted: specificity, sensitivity, positive and negative predictive value (PPV and NPV), false positive and false negative rates (FPR and NPR), and overall accuracy (OA).

## 3. Results

In total 1006 histologically confirmed cases (161 without evidence of CIN or malignancy, 510 CIN-1, 159 CIN-2, 129 CIN-3, and 47 cervical cancer cases (29 squamous cell carcinomas (SCC) and 18 adenocarcinomas (ADENO-CA))) were included in this study and additionally 619 CN cases. The correlation of the cytological versus the histological outcome of our material appears in Table 1. In our material, the percentage of valid data (i.e., after excluding inadequate, invalid, and unsatisfactory results) was for the cytological examination 96.25%, for ARRAYS 91.94%, for NASBA 67.75%, for flow cytometry 81.54%, and for p16 68.68%.

For the construction of the CART model, the CHAID algorithm was used; the CART architecture was 20-5-10; that is, each parent node was forced to 20 or more vectors, and each terminal node had more than 5 vectors, and the tree depth was not allowed to grow more than 10 levels. The system was pruned to obtain simpler forms, the significance level for splitting a node was set to 0.05, the chi-square statistic was based on the likelihood ratio and the significance values were adjusted using the Bonferroni method, resplitting of merged categories within one node was allowed, the age and flow percentage intervals were set to 10 (i.e., flow and age values had 10 levels), and finally the maximum number of iterations was 500.

**Figure 1:**
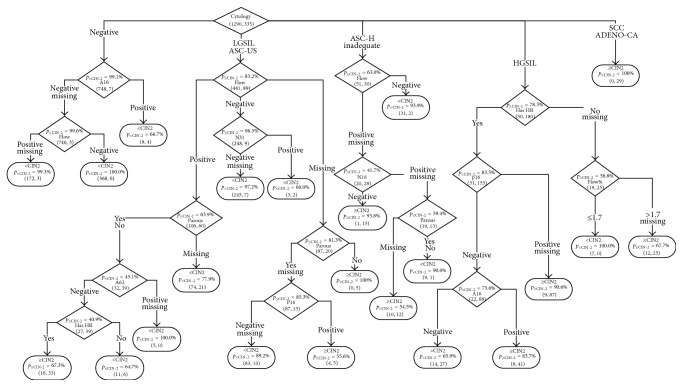
Structure of the CART model.

The CART architecture appears in Figure 1. It is worth noting that the role of the cytological examination result is dominant and that characteristics, such as the woman's age and the majority of HPV subtypes as were identified by the HPV subtyping test, were not found significant to be included. An example of a method of tree usage in practice is as follows.

- The user starts from the top node and examines the value of the proposed characteristic, in our case the cytological examination outcome.
- According to the value the user navigates to the appropriate node; in our case if the cytological examination is inadequate, we examine the flow result and a negative result leads the user to a probability for the case to be benign equal to 93.9% while if there is no result or the outcome of flow cytometry is positive then the user may examine the existence on HPV subtype 16 within the mRNA test, and interestingly a negative result provides a probability for malignancy equal to 93.8% as 15 out of the 16 cases with this profile were ≥CIN2 in histology.
- During navigation to the tree, the user may choose to stay in the proposed node if he/she is satisfied by the risk danger (probability) otherwise may examine the next proposed feature to find a more accurate result.
- The steps are repeated down to the terminal nodes if the user is not satisfied from the proposed risk levels from the previous parent nodes.

In a second example, the user may start with a negative cytological examination result; according to the lab performance, the probability for such a case to be positive is very small (0.9%). However, if more guarantees are required for this result, an HPV DNA test may be performed, a positive outcome on HPV subtype 16 reduces the probability of a case to be less than CIN-2 from 99.1% to 66.7% (4 out of the 12 cases with negative cytology and positive HPV DNA subtype 16 were finally found histologically CIN-2 or worst), and a negative or invalid result on test for HPV subtype 16 combined with a negative result for flow cytometry practically assures the woman that the probability to have a lesion worse than CIN-2 is negligible, as 568 cases with this profile in our material had less than CIN-2 and no case was found with equal or more than CIN-2 lesion.

A third example is related to the cases that are ASC-US or LGSIL in cytology; if the triage is based only on the cytological examination, all these women should be referred to colposcopy. However, in our material 441 cases were <CIN2 and 89 were ≥CIN2 (in total 530 women); thus, the probability to have <CIN2 is 83.2%, and if additional material is available in the vial, then a flow examination may be more helpful for the triage; in particular, a negative result in the flow examination gives a probability for <CIN2 equal to 96.5%, while a positive result indicates that this woman has more chances to harbor ≥CIN2 (46.1% Figure 1), and in such case these women could be immediately referred to colposcopy. This approach would allow reduction of referrals to colposcopy by 257, in more detail from 530 to 273; therefore, about half of the women (51.51%) could avoid immediate referral to colposcopy.

The assignment matrix of CART results appears in Table 3; actually the system classified 1216 out of the 1290 <CIN2 cases and 279 out of the 335 ≥CIN2 cases, and the statistics show that the model had sensitivity: 83.28%, specificity: 94.26%, PPV: 79.04%, NPV: 95.60%, FPR: 5.74%, FNR: 16.72%, and OA: 92.00%. In order to assess the CART performance in relation to the performance of each individual examination, the related statistics were extracted.

Specifically in Table 4 are summarized the performance metrics of the CART model, of the cytological examination using the ASC-US cases as a cutoff (i.e., all cases that were ASC-US and above in cytology were considered positive and referred to colposcopy) and similarly using an ASC-H cytological outcome as cutoff. For the HPV DNA typing, three alternative methods were evaluated: a woman was considered to be at risk and referred to colposcopy if (1) any HPV type was found, (2) only if a high risk type was found, and (3) only if 16 or 18 subtypes were identified. Finally, in Table 4 are presented the performance metrics for mRNA test using NASBA or flow cytometry and p16; that is, a case was considered to be at risk if any subtype was found in the NASBA examination or if a flow cytometry result was positive or screening of a p16 slide gave a positive result.

Finally in order to allow a more detailed evaluation of the methodology, in Table 5 are summarized the results of the histological outcome (blocks of rows) along with the cytological categories to which the cases were assigned (rows) in combination with the CART model outcome (columns).

## 4. Discussion and Conclusions

Test Papanicolaou is viewed as the most successful CC test [43] if it is repeatedly applied. However, CC is not yet eliminated even in countries with well-organized cervical cancer screening programs. There are many available options for the application of biomarkers in the triage of abnormal cases [17, 44–51]; however, these are either highly sensitive or highly specific, but not both at the same time. Nowadays, there is no consensus for the optimal management of women with abnormal Pap smears and equally not infrequently women with negative cytology are found to have a high-grade lesion ≥CIN2 histologically. Women with an ASC-US result in cytology present more complex management problems. The widely accepted management options of such cases are either immediate referral to colposcopy or surveillance with repeated Pap tests. The first option can overload colposcopy clinics and may lead to overintervention and overtreatment due to subtle findings. Overtreatment commonly has negative psychological effects with increasing anxiety and may further increase the risk for long-term perinatal morbidity in subsequent pregnancies [52]. Conversely, repeat cytology with surveillance has an inherent risk of missing HGSILs, dependent on the laboratory performance, has the risk of poor compliance, and may inversely increase the psychological burden for women with cytological abnormalities that are not further assessed. It is clear that we need more accurate diagnostic tools in order to limit the number of unnecessary colposcopic referrals without compromising the detection of high-grade disease.

In our material the percentage of ≥CIN2 cases in the total of the cases given as ASC-US was 23/169 = 13.61%. Furthermore, the percentage of ≥CIN2 cases in the total of LgSIL cases was 66/361 = 18.28%. Both percentages are in agreement with those reported by other researchers [53] that range between 5–17% and 9–16%, respectively, in the published literature. On the other hand, the percentage of cases given in cytology as HGSIL and found histologically lower than CIN2 was 50/230 = 21.74%. This is also consistent with the rates published in the literature [54, 55], demonstrating an agreement across various study settings.

Exploitation of ancillary test data for improvement of cervical intraepithelial lesions is nowadays a hot research topic with important applications. Since 2010, the Hellenic Cervical Pathology (HeCPA) Study Group is working on innovative approaches that use advanced mathematical and computing tools for the exploitation of ancillary tests that are nowadays available. Up to now, preliminary results are presented in the literature [35, 56]. In our previously published study [35], we applied CART models based on a smaller dataset, using cases that had valid examination results for all the available ancillary tests. This approach had clearly the disadvantage of a reduced usable data volume and does not capture the real life situation, that is, missing values. In addition, parameters related to woman history and demographic data were not included and the probabilities for a woman to harbor CIN were not calculated for each tree part. In two other published reports [56, 57] by the same group, there were applied neural networks to solve the same problem; the disadvantage of these approaches was again the requirement to have complete data for each series and no risk estimation was performed. In this study, we exploited the CART ability to handle cases with missing data and therefore increase the power of the study. The probability for each individual part of the tree was extracted. We used additional information related to women and concluded that parous is an important factor. We also extracted knowledge from our dataset in the form of triage algorithms that not only could be useful to the decision-makers towards their requests for ancillary tests but also promote a scoring system classifying individual women as high, low, or middle risk.

According to our results, despite the multitude of features entered into the CART model (Table 2), the training algorithm identified as useful only a small number of those parameters and was finally included in the CART model (Figure 1). The major discriminating characteristic was the cytological diagnosis; in relation to typing, only the existence of any high risk and of individual subtypes 16 and 62 was found important in our dataset and especially subtype 62 was a discriminating factor for a small number of cases. In relation to E6 and E7 expression, it was found that the flow cytometry results expressed both as positive/negative and as a percentage as well as the subtype 31 from the NASBA examination were important. Moreover, the immunocytochemical expression of p16 and parous data also appeared in the CART branches.

Based on the results, the proposed methodology had superior performance in relation to the overall accuracy (92.00%) than the majority of alternative methods (Table 4). There was marginal statistically significant difference only between CART and the cytology with ASC-H+ threshold (*χ* ^2^ = 4.027, *P* = 0.0448). However, for all other comparisons, the differences in the overall accuracy were statistically significant, specifically CART against cytology with ASC-US+ threshold (*P* < 0.0001), arrays using any type (*P* < 0.0001), arrays using high risk subtypes (*P* < 0.0001), arrays for subtype 16 or 18 (*P* < 0.0001), NASBA for any type (*P* < 0.0001), and finally p16 (*P* < 0.0001). In relation to the comparison of the CART model and the cytological examination with threshold ASC-H+, the sensitivity of the CART model (83.28%) was significantly higher than cytology (69.33%); therefore, the proposed method had significantly (*P* < 0.0001) better performance than all other alternatives.

In relation to the false positive cases, the CART wrongly categorized 74 cases as positive (≥CIN2); from these 12 were negative and 62 CIN-1 in histology, although 50 of these cases were given as ASC-H or HGSIL in cytology (Table 5), 17 as LGSIL, 3 as ASC-US, and the remaining 4 as inadequate in cytology. No case was cytologically negative, as the cytological result is the primary characteristic that is considered as important by our methodology, and these results were expected. The FPR of the CART model was 5.74%, outperformed only by the cytological result with ASC-US+ cutoff (2.24%) but at the cost of specificity (94.26 versus 59.79%; see Table 4).

The analysis of false negative cases is more important; the CART model gave 56 false negative cases in total (14 ASC-US, 32 LGSIL, 2 inadequate, 7 negative in cytology, and 1 ASC-H; see Table 5). None of these cases had HGSIL or cancer as cytological result. The histological outcome of these cases was 41 CIN-2, 13 CIN-3, and 2 adenocarcinomas. The cytological result for the last 2 cases was ASCH and LGSIL and there was additionally colposcopical agreement.

In relation to the 61 samples that were inadequate in cytology (Table 5), 22 were ≥CIN2, among them 4 carcinomas (2 adenocarcinomas and 2 SCC), 9 CIN-2, and 9 CIN-3, and the remaining 39 cases were <CIN2; the CART model classified correctly 54 of them (35 <CIN2, 8 CIN-2, 8 CIN-3, 2 SCC, and 2 adenocarcinomas) and missed 6 cases (4 CIN-1, 1 CIN-2, and 1 CIN-3). It is worth noting that this decision was based only on biomarker data and parous status. Therefore, the number of women that would require a second cytological examination could be reduced dramatically.

Concluding the application of the proposed method gave encouraging results and not only could be helpful towards a better management of women for various findings during cytological examination but also provides a flexible technique for the estimation of ≥CIN2 risk. As a result, the proposed method provides a guide towards personalized management and therapeutic decisions, may reduce the overload of colposcopy clinics and unnecessary treatments, and identifies a higher percentage of women at risk of cervical cancer or precancerous lesions.

## Figures and Tables

**Table 1:** Correlation of the cytological with the histological diagnosis.

	Histological result							Subtotal
	CN	Negative	CIN 1	CIN 2	CIN 3	SCC	ADENO-CA	
Cytology								
Inadequate	—	10	29	9	9	2	2	61
Negative	619	62	67	5	2	—	—	755
ASC-US	—	37	109	18	5	—	—	169
LGSIL	—	36	259	50	15	—	1	361
ASC-H	—	6	6	2	3	2	1	20
HGSIL	—	10	40	75	94	9	2	230
SCC	—	—	—	—	1	15	2	18
ADENO-CA	—	—	—	—	—	1	10	11

Total	619	161	510	159	129	29	18	1625

**Table 2:** Variables entered to the model.

Variable name	Description	Value range
Cytology	The result of the cytological examination expressed according to the Bethesda 2009 system	WNL, ASC-US, LGSIL, ASC-H, HGSIL, SCC, ADENO CA, and <blank> if there is no result

HPV DNA arrays A6, A11, A16, A18, A26, A31, A33, A35, A39, A40, A42, A43, A44, A45, A51, A52, A53, A54, A56, A58, A59, A61, A62, A66, A68, A70, A71, A72, A73, A81, A82, A83, A84, A85, and A89	The existence of individual subtypes according to the arrays examination	0 if the specific subtype is not found 1 if the specific subtype is found <blank> if there is no result

Has HR	Positive if one or more high risk subtypes were found during typing	Positive, negative, or missing

Has LR	Positive if one or more low risk subtypes were found during typing	Positive, negative, or missing

Has any type	Positive if one or more subtypes were found during typing	Positive, negative, or missing

No HR	The number of high risk subtypes that were found during typing	An integer or missing

No LR	The number of low risk subtypes that were found during typing	An integer or missing

N16, N18, N31, N33, and N45	The result of the E6/E7 mRNA test for the specific HPV subtype	0 if negative 1 if positive <blank> if there is no result

Flow	The result of the identification of E6/E7 mRNA expression of high risk HPV using flow cytometry technique	0 if negative (<1.5%) 1 if positive (>1.5%) <blank> if there is no result

Flow %	The result of E6/E7 mRNA expression of high risk HPV using flow cytometry technique expressed as a percentage	A number or <blank> if there is no result

p16	The result of the p16 immunocytochemical examination	0 if negative 1 if positive <blank> if there is no result

Age	The woman age at the time of examination	A positive number

Parous	Woman parous status	1 if she has born one or more children and 0 if not

**Table 3:** CART results.

Actual category	Predicted category		Grand total
	<CIN 2	≥CIN 2	
<CIN 2	1216	74	**1290**
≥CIN 2	56	279	**335**

Grand total	**1272**	**353**	**1625**

**Table 4:** Performance of CART and individual examinations.

	CART	Cytology using cutoff ASC-US+	Cytology using cutoff ASC-H+	Arrays (positive if any type was found)	Arrays (positive if a high risk type was found)	Arrays (positive if subtype 16 or 18 was found)	NASBA (positive if any type was found)	Flow cytometry (positive if the result is positive >1.5%)	p16
Sensitivity	83.28%	97.76%	69.33%	87.71%	84.72%	52.82%	69.65%	88.19%	57.21%
Specificity	94.26%	59.79%	95.04%	70.49%	75.02%	90.28%	87.09%	79.60%	93.57%
PPV	79.04%	37.82%	77.78%	42.86%	46.11%	57.82%	62.15%	48.49%	69.68%
NPV	95.60%	99.07%	92.53%	95.79%	95.11%	88.35%	90.41%	96.87%	89.44%
FPR	5.74%	40.21%	4.96%	29.51%	24.98%	9.72%	12.91%	20.40%	6.43%
FNR	16.72%	2.24%	30.67%	12.29%	15.28%	47.18%	30.35%	11.81%	42.79%
OA	92.00%	67.39%	89.90%	73.96%	76.97%	82.73%	83.02%	81.13%	86.11%
Valid results	**1625**	**1564**	**1564**	**1494**	**1494**	**1494**	**1101**	**1325**	**1116**
% of valid results	**100.00%**	**96.25%**	**96.25%**	**91.94%**	**91.94%**	**91.94%**	**67.75%**	**81.54%**	**68.68%**

**Table 5:** CART results in relation to the cytological outcome and the histological result.

Histology	Cytology	CART result			Grand total
		Correct	False negative	False positive	
CN	Negative	619			**619**

(CN) Total		**619**			**619**

Negative	Inadequate	10			**10**
	Negative	62			**62**
	ASC-US	36		1	**37**
	LGSIL	36			**36**
	ASC-H	3		3	**6**
	HGSIL	2		8	**10**

Negative total		**149**		**12**	**161**

CIN 1	Inadequate	25		4	**29**
	Negative	67			**67**
	ASC-US	107		2	**109**
	LGSIL	242		17	**259**
	ASC-H	2		4	**6**
	HGSIL	5		35	**40**

CIN 1 total		**448**		**62**	**510**

CIN 2	Inadequate	8	1		**9**
	Negative		5		**5**
	ASC-US	7	11		**18**
	LGSIL	26	24		**50**
	ASC-H	2			**2**
	HGSIL	75			**75**

CIN 2 total		**118**	**41**		**159**

CIN 3	Inadequate	8	1		**9**
	Negative		2		**2**
	ASC-US	2	3		**5**
	LGSIL	8	7		**15**
	ASC-H	3			**3**
	HGSIL	94			**94**
	SCC	1			**1**

CIN 3 total		**116**	**13**		**129**

SCC	Inadequate	2			**2**
	ASC-H	2			**2**
	HGSIL	9			**9**
	SCC	15			**15**
	ADENO-CA	1			**1**

SCC total		**29**			**29**

ADENO-CA	Inadequate				**2**
	LGSIL		1		**1**
	ASC-H		1		**1**
	HGSIL	2			**2**
	SCC	2			**2**
	ADENO-CA	10			**10**

ADENO-CA total		**16**	**2**		**18**

Grand total		**1495**	**56**	**74**	**1625**
